# A quality by design HPLC method for cephalosporin analysis in pharmaceuticals and water samples with environmental impact assessment

**DOI:** 10.1038/s41598-024-84647-y

**Published:** 2025-01-02

**Authors:** Ali Alqahtani, Taha Alqahtani, Adel Al Fatease, Enas H. Tolba

**Affiliations:** 1 Department of Pharmacology, College of Pharmacy, King Khalid University, Abha, 62529 Saudi Arabia; 2 Department of Pharmaceutics, College of Pharmacy, King Khalid University, Abha, 62529 Saudi Arabia; 3 Egyptian Drug Authority (EDA), Giza, 35521 Egypt

**Keywords:** Cephalosporins, Green chemistry, HPLC, Box–Behnken, Analytical chemistry, Green chemistry

## Abstract

The present study applied a combined analytical quality-by-design and green analytical chemistry approach to develop an HPLC method for the determination of four cephalosporin pharmaceuticals in both their formulations and water samples. These drugs include ceftriaxone, cefotaxime, ceftazidime and cefoperazone. A Box–Behnken experimental design was employed to optimize three chromatographic parameters: mobile phase composition, flow rate and buffer pH. The predicted optimal conditions involved using a mobile phase of acetonitrile and 0.04 M phosphate buffer at pH 6 in a 7:93 (v/v) ratio, pumped at 1.3 mL/min through a Nucleosil C18 (4.6 × 250 mm, 5 μm) column with UV detection at 240 nm. Under these optimum conditions, the developed HPLC method successfully separated the four drugs with good resolution in less than 6 min. Linearity was established across the concentration ranges of 5–300 µg/mL for ceftriaxone and cefotaxime, 5–400 µg/mL for ceftazidime and 5–100 µg/mL for cefoperazone. Furthermore, full validation of the method in terms of accuracy, precision, specificity and robustness was carried out as per ICH guidelines. The greenness profile of the optimized HPLC method was also evaluated using the Analytical GREEnness (AGREE) tool and found to be environmentally friendly with AGREE score of 0.75, making it a greener alternative for quality control and routine analysis of the investigated cephalosporins in their pharmaceutical formulations and tap water samples. Furthermore, the blueness assessment of the proposed HPLC method using the blue applicability grade index (BAGI) tool yielded a value of 77.5, indicating its high analytical practicality and substantial potential for routine analysis applications.

**Supplementary Information:**

The online version contains supplementary material available at 10.1038/s41598-024-84647-y.

## Introduction

β-Lactam antibiotics are a crucial class of antimicrobial drugs, which have been extensively used to treat a wide range of bacterial infections^1^. These antibiotics contain a β-lactam ring in their molecular structure and can be categorized into penicillins, cephalosporins, monobactams, and carbapenems based on their chemical composition^2^. Among β-lactam antibiotics, cephalosporins are widely used in clinical settings for the treatment of various bacterial infections. Cephalosporins inhibit the synthesis of the peptidoglycan layer of the bacterial cell wall and are valued for their broad spectrum activity against both Gram-positive and Gram-negative bacteria, making them important therapeutic agents in fighting infectious diseases^3^. Their significance lies in effectively combating bacterial pathogens, including those that have developed resistance to other antibiotic classes^3^. However, excessive use and improper utilization particularly in less developed countries can lead to environmental contamination as well as spread antibiotic resistance genes^4^. Therefore, monitoring and controlling antibiotic contamination is crucial for safeguarding health and maintaining ecological balance. All of this emphasizes the need for comprehensive analytical methods to monitor and control the presence of these antibiotics in the environment.

A literature review revealed the determination of cephalosporins in various matrices through different analytical techniques including UV spectrophotometry^5–9^, high performance liquid chromatography (HPLC)^10–16^, ultra performance liquid chromatography (UPLC)^17^, high performance thin layer chromatography (HPTLC)^18^ and capillary electrophoresis (CE)^19–21^. In addition, official pharmacopeial chromatographic methods have been reported for the determination of the four drugs under investigation^22^. However, the majority of the reported methods including the pharmacopeial methods employ complex sample preparation, use of hazardous organic solvents, time wasting and lack of greenness.

Analytical Quality by Design (AQbD) is a risk-based approach to analytical method development, emphasizing the importance of incorporating quality into the procedure from the outset^23^. This design of experiments technique utilizes minimal experiments to analyze factors and their interactions on measured responses, ultimately achieving desired outcomes^24,25^. Additionally, AQbD serves as a pre-validation methodology aiming to establish a comprehensive framework for method variables especially in chromatographic methods such as mobile phase composition, pH, column temperature, and flow rate^26,27^. Through systematic evaluation and optimization using experimental design, we can ensure that the chromatographic method effectively separates and quantifies analytes in pharmaceutical formulations while remaining robust and sensitive enough^28^.

Green chemistry is an approach that aims to reduce the environmental and human health impacts of chemical production and consumption. Green Analytical Chemistry (GAC) is a branch of green chemistry specifically dedicated to developing analytical techniques and methods that decrease or eliminate the use and generation of hazardous substances^29^. The aim is to make analytical processes more eco-friendly, while maintaining or enhancing their effectiveness^30^. This can be achieved by using safer chemicals and solvents, minimizing waste generation, reducing energy consumption, using small sample sizes and reagent volumes, as well as decreasing derivatization reactions^31^. Consequently, GAC collaborates with AQbD in creating a comprehensive approach for developing scientifically rigorous, cost-effective and environmentally sustainable analytical methods^32^. This collaboration has become increasingly crucial in today’s analytical laboratories. One of the most used metrics to quantitatively analyze the greenness of analytical method is the Analytical GREEnness metric (AGREE), which provides a comprehensive assessment of the environmental impact of the entire analytical process based on the 12 principles of green chemistry^33^. The analytical practicability of the developed method is also another critical aspect to consider. Recently, a new metric called the blue applicability grade index (BAGI) has been proposed to evaluate the usability and applicability of analytical methods i.e. method blueness^34^.

**Fig. 1:**
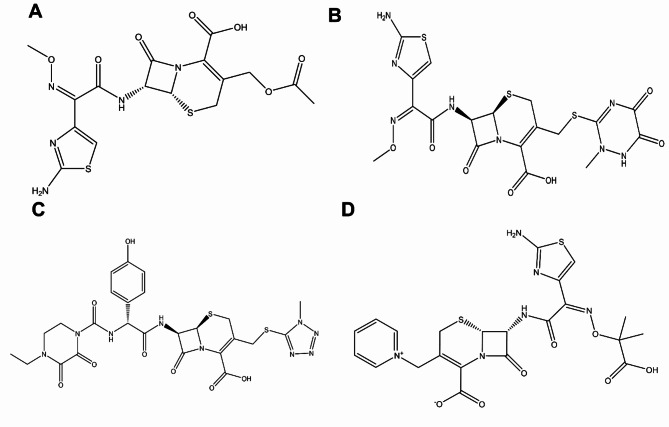
Chemical structures of (**A**) cefotaxime, (**B**) ceftriaxone, (**C**) cefoperazone and (**D**) ceftazidime.

The aim of the present work is the development and the validation of a green HPLC method for the simultaneous determination of four cephalosporin antibiotics (ceftriaxone, cefotaxime, ceftazidime, and cefoperazone) (Fig. 1) in their pharmaceutical formulations and water samples using an AQbD approach. While this method is not intended to replace established pharmacopeial methods for individual drug analysis in pharmaceutical quality control, where more rigorous validation criteria and specificity studies are required, it offers an efficient analytical approach for studies requiring monitoring of multiple cephalosporins, such as environmental water analysis and preliminary pharmaceutical screening. The developed method will be validated as per the ICH guidelines to ensure the accuracy, precision, specificity, linearity and robustness of the analytical procedure. Moreover, the greenness and blueness of this method will be evaluated to guarantee its environmental impact and analytical applicability.

## Experimental

### Materials and reagents

Cefotaxime (CFO), ceftriaxone (CFT), cefoperazone (CFP) and ceftazidime (CFZ) standards were provided by Sigma-Aldrich (St. Louis, MO, USA) with purities of 99.50%, 99.70%, 99.80% and 99.60%, respectively. HPLC-grade acetonitrile was supplied by Scarlau (Barcelona, Spain) while ortho-phosphoric acid was obtained from Fluka Analytical (Seelze, Germany). Potassium dihydrogen orthophosphate was acquired from Honeywell Riedel-deHaën (Seelze, Germany). Furthermore, pharmaceutical formulations including Claforan, Ceftriaxone, Cefozon and Fortum were procured from local pharmacies, Cairo, Egypt.

### Instrumentation and software

The development and the validation of the methods were performed using an Agilent 1260 liquid chromatography system. It was equipped with a quaternary pump, UV-vis detector, manual injector with a 20 µL loop and degasser. Chromatographic separation was carried out on Nucleosil C18 column (4.6 × 250 mm, 5 μm) from Macherey-nagel (Duren, Germany). The mobile phase was vacuum filtered through 0.45 μm nylon membranes before use. A Jenway 3510 Standard digital pH meter kit; 230 VAC/UK with a glass electrode was used to adjust the pH. Chemstation software (B.04.03) was used for instrumental control, data analysis and acquisition. The experimental design was created using Design-Expert software (7.0.0) from Stat-Ease, Inc.

### Preparation of standard stock solutions

An accurate weight of 50 mg of CFO, CFT, CFP and CFZ were separately measured and transferred into a series of 50 mL volumetric flasks. The contents of the flasks were dissolved using distilled water and filled to the mark to achieve CFO, CFT, CFP and CFZ stock solutions concentration at 1 mg/mL. For analysis purposes, newly prepared working solutions were made by diluting these stock solutions into mobile phase.

### Experimental design for optimizing chromatographic parameters

#### Defining analytical target profile and critical analytical attributes

To develop a reliable analytical technique for estimating drugs in their pharmaceutical formulations, the initial step involves accurately outlining the analytical target profile (ATP). This will form the basis for determining the main goals and expected results of the method. The ATP statement will direct the selection, design and advancement of the method, playing a crucial role in constantly enhancing the analytical techniques^35^. Moreover, it will aid in choosing appropriate analytical procedures after regulatory approval and assessing the method’s suitability during development as well as throughout its lifecycle^23^. The main goal of our approach was to create a precise and reliable analytical method that is also simple and sensitive. On the other hand, measurable variables reflecting method performance, known as critical analytical attributes (CAAs), were identified through initial experiments, with peak resolution and run time being the most significant CAAs.

#### Quality risk assessment and scouting phase

A risk-focused approach is essential as a key element of AQbD, in line with ICH Q8 and Q9 guidelines. The Ishikawa fishbone diagram, which is rooted in scientific principles and designed to generate ideas about all possible analysis parameters while identifying potential risks based on previous knowledge and experimentation, can be utilized for quality risk assessment^36^. Many parameters were studied to determine the overall risk to the performance of the method using the Ishikawa fishbone diagram, including the use of acetonitrile in small quantities as an organic modifier based on GAC principles in the organic phase and employing isocratic elution mode for its advantages such as simplicity, and no need for column re-equilibration between injections compared to gradient elution. Different stationary phases like C18, C8 and CN columns were evaluated, and preliminary results indicated that Nucleosil C18 column (4.6 × 250 mm, 5 μm) sufficiently retained and separated the compounds under study. A detection wavelength at 240 nm was chosen due to provide optimal sensitivity. In contrast, pH scouting studies were conducted in the range of 3.0–7.0 for the used C18 column to understand how drugs behave over different pH values. A pH from 4.0 to 6.0, revealed high resolution data with low tailing factor for all examined drugs.

#### Box–Behnken design for method optimization

Based on the initial findings of the scouting and the risk assessment stages, a Box–Behnken experimental design was utilized to optimize the method parameters and their interactions. A total of 15 experiments were carried out with 3 central point replicates to evaluate the effects and interactions of the following three independent variables: mobile phase composition (% acetonitrile), pH and flow rate (Table S1). The peak resolutions of A from B (R1), B from C (R2) and C from D (R3) were measured as responses R1, R2 and R3, respectively. Besides, chromatographic run time was tested as the fourth response (R4) in order to maximize greenness of the method. Linear and quadratic models were used to analyze the results and the quality of the fitted models was evaluated using the coefficient of determination and probability values. Derringer’s desirability function was applied to find the optimal condition.

### Procedure

#### Chromatographic procedure

The HPLC analysis was carried out using Nucleosil C18 column. The mobile phase of acetonitrile and 0.04 M potassium dihydrogen orthophosphate at pH 6.0 was pumped at a flow rate of 1.3 mL/min. Quantification was based on peak area with detection performed at 240 nm.

#### Method validation

Linearity of the developed method was evaluated by preparing calibration solutions of the four cephalosporin antibiotics: CFO, CFT, CFP and CFZ, at concentrations ranging from 5 to 300 µg/mL, 5–300 µg/mL, 5–100 µg/mL and 5–400 µg/mL, respectively. To prepare these solutions, different aliquots of the standard stock solutions were transferred into 10 mL volumetric flasks, which were then diluted to the mark with the mobile phase. Each solution was injected onto the HPLC column in triplicate, and the established chromatographic conditions were applied. Four separate calibration curves were constructed by correlating the peak area with the corresponding analyte concentration. The regression equations and the r^2^ values for each analyte were derived. The limits of detection (LODs) and quantification (LOQs) were calculated based on the standard deviation of response and the slope of the calibration curve. The accuracy and precision of the method were evaluated through analyzing triplicate samples at three different concentration levels (80%, 100% and 120% of the nominal analytes’ concentration). The nominal concentrations of samples were 150 µg/mL, 150 µg/mL, 50 µg/mL and 200 µg/mL for CFO, CFT, CFP and CFZ, respectively. The results were expressed as RSD% for intra-day and inter-day precisions and % recoveries for accuracy.

Specificity of the method was examined by standard addition to pharmaceutical formulations containing the target analytes. Method robustness was evaluated by observing the effects of small deliberate variations in critical method parameters, such as: mobile phase pH (± 0.2), flow rate (± 0.1), and organic solvent composition (± 1%) and the %recovery ± RSD% were calculated.

#### Analysis of CFO, CFT, CFP and CFZ in their dosage forms and tap water samples

The suitability of the proposed HPLC method for quantifying the cephalosporin antibiotics in their pharmaceutical formulations was evaluated. The contents of five vials labeled to contain 1000 mg of the analytes were weighed and the mass of the powdered commercial formulation per vial was calculated to be 100 mg for each drug. These samples were dissolved in 100 mL of distilled water to prepare stock solutions. Subsequent dilutions were made in the mobile phase to obtain different concentrations for each cephalosporin. The prepared samples were filtered through a 0.45 μm membrane filter prior to injection into the chromatographic system. The amounts of CFO, CFT, CFP and CFZ in the samples were determined using their respective calibration curves.

The developed HPLC method has also been employed to determine the studied analytes in spiked tap water samples to investigate their applicability for analyzing the antibiotics in environmental matrices. Five milliliters of water samples were spiked with working standard solutions of CFO, CFT, CFP and CFZ to achieve final concentrations covering the linearity range for each drug. These samples were then analyzed using the developed HPLC procedure for the simultaneous determination of these antibiotics in the spiked water samples.

## Results and discussion

The primary aim of this work was to develop a simple, rapid, green, and validated HPLC method for the simultaneous determination of four cephalosporin antibiotics, including CFO, CFT, CFP and CFZ in their pharmaceutical formulations and tap water samples. The main challenge was to find the optimal chromatographic conditions to achieve their separation with a high resolution factor (Rs > 1.5), satisfactory peak shapes and appropriate retention times, as well to minimize the use of organic modifier during the analysis process in order to enhance the method’s environmental friendliness. This can be accomplished by adjusting key chromatographic parameters such as buffer pH, acetonitrile percentage and mobile phase flow rate via AQbD approach.

### Box–Behnken design to optimize chromatographic parameters

The design was developed using Design-of-Experiments technique known as the Box–Behnken design (BBD) as shown in Table S1, which helps identify the chromatographic parameters for achieving optimal separation with fewer experimental trials and time investment. Using BBD is advantageous when optimizing chromatographic procedures for analysis, particularly in significantly reducing the required experiments. However, it’s important to note that the BBD does not cover all possible combinations of variables at extreme levels such as highest or lowest, so evaluations at those magnitudes may result in suboptimal outcomes. The chosen parameters for investigation were buffer pH, % acetonitrile, and mobile phase flow rate, while the resolution between each peak pair was set as response variables in addition to the total run time. Multiple linear regression analysis on the data obtained from the design matrix yielded polynomial equations correlating the responses to the tested variables as follows:

1 $$\text{R}1=+14.37-1.41\times\text{A}-2.45\times\text{C}-1.51\times\text{C}2$$

2 $$\text{R}2=+3.33-0.97\times\text{A}-1.64\times\text{C}$$

3 $$\text{R}3=+14.18-1.67\times\text{A}-3.14\times\text{C}-1.68\times\text{C}2$$

4 $$\text{R}4=+6.11-0.75 \times \text{A}-0.98\times \text{C}$$

**Fig. 2:**
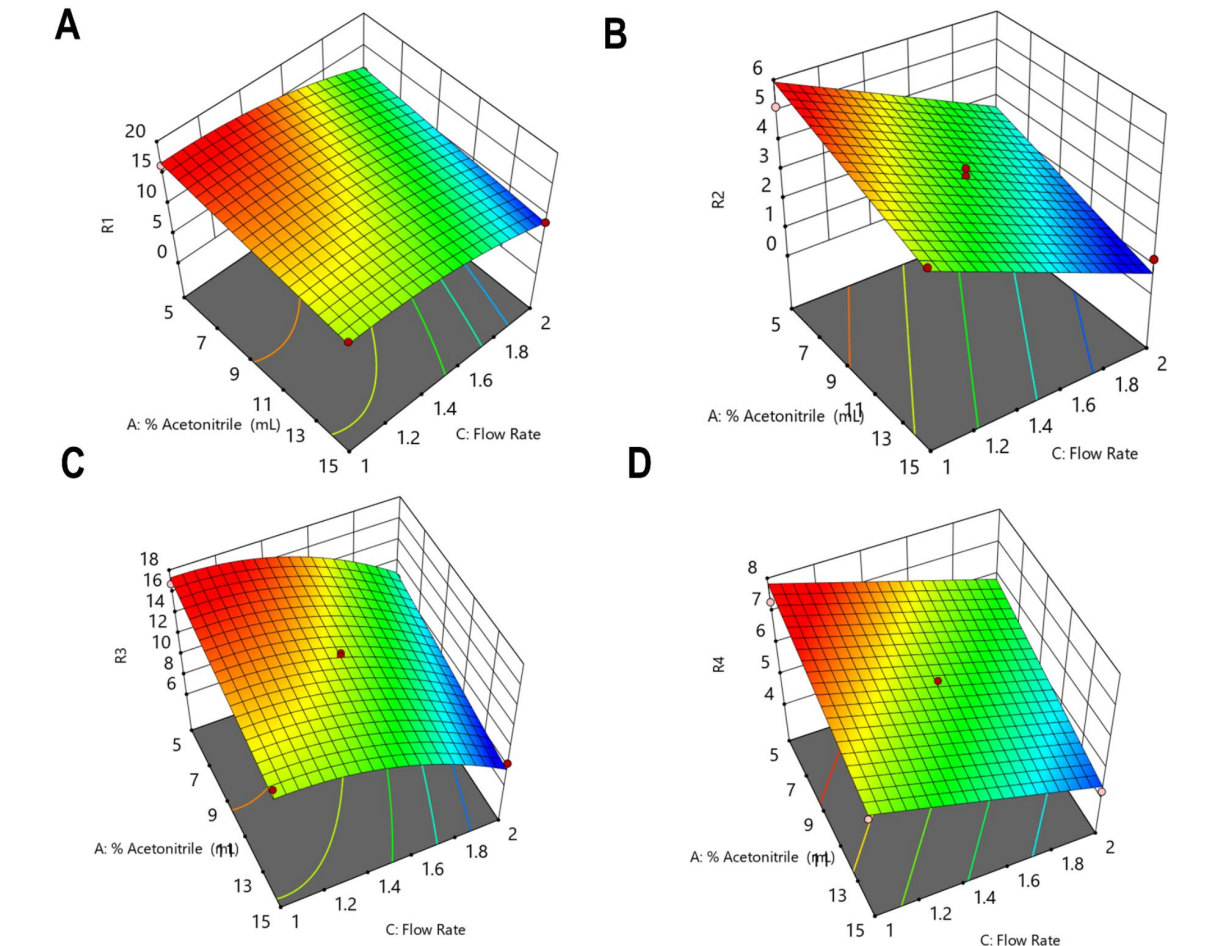
3D-response surface plots showing the effects of % acetonitrile and flow rate on (**A**) the resolution between ceftriaxone and cefotaxime, (**B**) the resolution between cefotaxime and ceftazidime, (**C**) resolution between ceftazidime and cefoperazone and (**D**) chromatographic run time. Plots were generated using Design-Expert software (version 7.0.0).

**Fig. 3:**
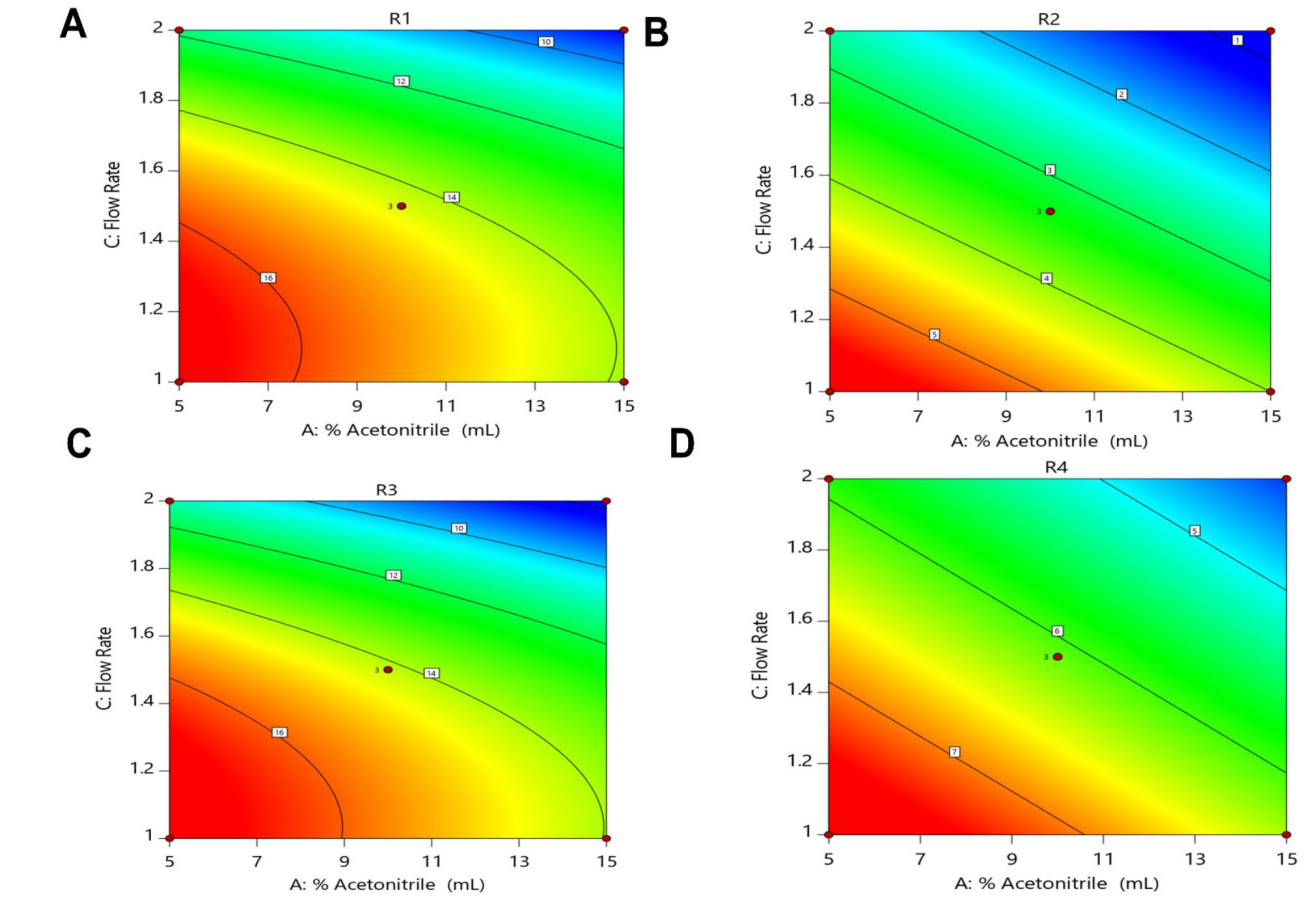
Contour plots showing the effects of % acetonitrile and flow rate on (**A**) the resolution between ceftriaxone and cefotaxime, (**B**) the resolution between cefotaxime and ceftazidime, (**C**) resolution between ceftazidime and cefoperazone and (**D**) chromatographic run time. Plots were generated using Design-Expert software (version 7.0.0).

Where, A is Acetonitrile %, C is flow rate and R1, R2 and R3 are the resolution responses between CFT and CFO, resolution responses between CFO and CFZ and resolution responses between CFZ and CFP, respectively, while R4 is the analysis time. From the previous regression analysis, second-order equations determine the curvature in the relationship among the response variables (R1 and R3) and the predictor variables while linear models could model the responses (R2 and R4). Considering the linear terms in all equations, it is noticed that % acetonitrile and flow rate both are dominant in influencing peak resolutions and analysis time, while pH of the buffer is not as affected as the other two variables. The negative sign of coefficients in all the regression equations indicates that the R1, R2 and R3 responses are inversely proportional to the flow rate and % acetonitrile. The negative sign of coefficient in (Eq. 4) indicate that the analysis time is decreasing as the % acetonitrile and flow rate are increased which suggest the importance of accommodation between these two factors in order to achieve the ideal resolutions between the peaks. While consuming fewer amount of the organic modifier is to enhance the greenness goal of the developed analytical method. The graphical representation of the effects of acetonitrile percentage and flow rate on the resolution factors (R1, R2 and R3) and analysis time (R4) is depicted through three-dimensional surface plots and two-dimensional contour plots, as shown in Figs. 2 and 3.

The models were analyzed using the analysis of variance test (ANOVA), and the results are shown in Tables S2–S5. The models were considered statistically significant as the p-values were lower than the significance level of 0.05 for all the responses. Interestingly, all the models were significant where the flow rate and % acetonitrile were the two most influential factors with p values < 0.05. The goodness of fit was evaluated through R^2^ and adjusted R^2^ values and all the values were greater than 0.8, indicating that the models explain more than 80% of the variability in the responses (Table S6). Additionally, the predicted R^2^ and the adjusted R^2^ values for all the responses showed a good agreement, confirming the adequacy of the models to navigate the design space. Residual plots play a vital role in assessing the goodness of fit of regression and ANOVA models by evaluating the patterns of residuals, their distribution, and any potential outliers or trends. The results of the residual analysis demonstrated that the residuals exhibited a normal distribution without any obvious patterns or outliers and maintained a constant variance across the predicted values as shown in (Fig. S1).

**Fig. 4:**
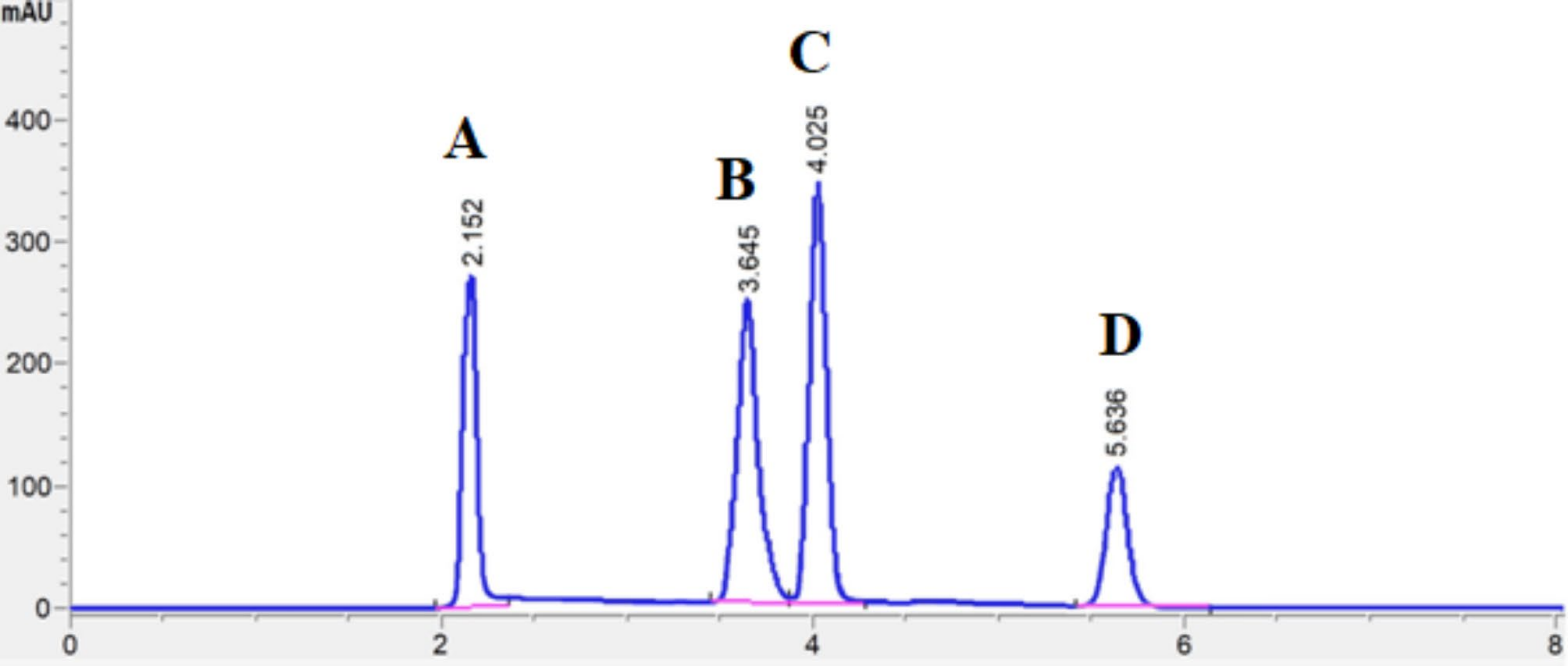
HPLC chromatogram corresponding to simultaneous analysis of CFT (**A**), CFO (**B**), CFZ (**C**) and CFP (**D**). Mobile phase: acetonitrile—0.04 M potassium phosphate buffer (pH 6) (7: up to 100, v/v); stationary phase: Nucleosil C18 (4.6 × 250 mm, 5 μm); flow-rate: 1.3 ml/min; temperature: ambient; detection: UV at 240 nm.

Numerical optimization was then performed to maximize the resolution factors and minimize the chromatographic run time simultaneously. The optimization desirability function combined all the responses into a single value, which was then maximized to find the optimal conditions. Based on Derringer’s desirability function, the optimum conditions were determined to be as the following: buffer pH at 6, % acetonitrile at 7% and flow rate at 1.3 mL/min. These conditions were capable to achieve baseline separation of the four cephalosporins, while consuming lower amount of the organic modifier during the analysis process, thus enhancing the greenness of the developed analytical method (Fig. 4).

### Method validation

After optimizing the chromatographic parameters using the Box–Behnken Design and selecting the most favorable conditions, an evaluation of system suitability parameters was conducted to ensure the HPLC system’s functionality prior to method validation (Table 1). Results indicate satisfactory system suitability parameters in terms of plate number, tailing factor, and peak resolution.

**Table 1:** System suitability tests of the proposed RP-HPLC method for the simultaneous determination of CFT, CFO, CFZ and CFP.

Parameter	CFT	CFO	CFZ	CFP
Number of theoretical plates (N)	9546	9110	11,458	6547
Resolution factor (R)	15.57^a^	4.26^b^	14.96^c^	–
Tailing factor (T)	0.93	0.868	0.907	0.907
Capacity factor (K)	1.152	2.645	3.025	4.636
Selectivity factor (α)	2.29	1.14	1.53	–
%RSD > of tr^D^ of 6 injections	0.55	0.64	0.54	0.71

^a^Resolution between CFT and CFO.

^b^Resolution between CFO and CFZ.

^c^Resolution between CFZ and CFP.

^D^Retention time (min).

The developed method was also validated according to ICH guidelines^37^ in terms of linearity, range, accuracy, precision, LODs, LOQs and specificity (Tables 2 and 3). Linearity was assessed in triplicates through the analysis of seven different concentrations of CFO, CFT, CFZ and eight different concentrations of CFP. High R^2^ values of 0.9999 confirmed strong linearity between peak areas and drug concentrations. Table 2 presents the slopes, intercepts, and regression coefficients for the four calibration curves along with LODs and LOQs of the studied analytes.

**Table 2:** Linearity results obtained by the proposed RP-HPLC method for the simultaneous determination of CFT, CFO, CFZ and CFP.

Item	CFT	CFO	CFZ	CFP
Retention time (tr) (min)	2.15 ± 0.07	3.65 ± 0.24	4.03 ± 0.17	5.64 ± 0.15
Wavelength of detection (nm)	240	240	240	240
Range of linearity (µg/mL)	5–300	5–300	5–400	5–100
Slope	25.992	26.014	57.096	23.596
Intercept	0.681	0.611	47.93	0.235
Regression coefficient (R^2^)	0.9999	0.9999	0.9999	0.9999
LOD (µg/mL)	1.530	1.640	1.605	1.594
LOQ (µg/mL)	4.635	4.972	4.864	4.829

The accuracy and precision of the proposed method was evaluated by analyzing three different concentrations representing 80%, 100% and 120% of the theoretical concentrations of CFO, CFT, CFP and CFZ, each point in triplicate. The percentage recoveries calculated for each drug demonstrated the high accuracy of the developed method, as shown in Table 3. The low values of RSD% (less than 2%) for both intra-day and inter-day precisions, confirm the high precision of the proposed analytical procedure (Table 3).

**Table 3:** Results for the determination of accuracy, intraday and interday precision for CFT, CFO, CFP, and CFZ, by the proposed RP-LC method:

Drug	Concentration (µg/mL)	Accuracy % Recovery ± SD	Intraday precision %RSD	Interday precision %RSD
CFT	120	99.56 ± 1.140	1.145	1.287
	150	100.09 ± 1.123	1.122	1.222
	180	100.24 ± 1.107	1.104	1.342
CFO	120	100.47 ± 1.138	1.133	1.241
	150	100.22 ± 1.157	1.154	1.335
	180	99.87 ± 1.144	1.145	1.317
CFZ	160	98.88 ± 1.089	1.101	1.211
	200	99.87 ± 1.136	1.137	1.345
	240	99.74 ± 1.198	1.201	1.307
CFP	40	100.71 ± 1.004	0.997	1.111
	50	100.92 ± 0.992	0.983	1.054
	60	100.37 ± 0.877	0.874	1.008

Specificity is the ability of the analytical method to measure the analyte response in the presence of interferences. The results indicate the absence of any interfering peaks at the retention times of the studied analytes, with recovery values around 100%, which meeting the acceptable criteria, thus demonstrating the specificity of the developed method (Table 4). Besides, standard addition technique was applied to check the method specificity and to assess any potential matrix effects in the drug product. Results indicated that there was no significant matrix effect, confirming no interference of the formulation excipients on the determination of the target analytes (Table 4). The robustness of the developed method was further assessed by deliberately modifying specific chromatographic parameters, such as the mobile phase pH, flow rate and organic solvent composition. These intentional variations in the experimental conditions did not significantly impact the chromatographic resolution of the studied analytes or their recovery values, as shown from RSD% values in Table S7.

**Table 4:** Specificity study of the proposed method using standard addition technique.

Drug	Pharmaceutical taken (µg/mL)	Pharmaceutical found (µg/mL)	Pure added (µg/mL)	Pure found (µg/mL)	%Recovery
CFT	150	148.77	15	15.10	100.67
			30	30.22	100.73
			45	44.80	99.56
Mean ± %RSD					100.32 ± 0.657
CFO	150	149.91	15	15.22	101.47
			30	29.88	99.60
			45	44.77	99.49
Mean ± %RSD					100.19 ± 1.111
CFZ	200	200.10	20	20.31	101.55
			40	40.54	101.35
			60	59.88	99.80
Mean ± %RSD					100.90 ± 0.949
CFP	50	49.90	5	5.02	100.40
			10	10.11	101.10
			15	15.16	101.07
Mean ± %RSD					100.86 ± 0.392

### Greenness and blueness assessment of the developed method

Greenness assessment of the proposed HPLC method was conducted using in comparison with the reported literature^16^ for the determination of CFO, CFT, CFP and CFZ to evaluate the environmental impact of the developed analytical method. Although our method and the reported method were optimized using design of experiment approaches, the development of the current environmentally friendly HPLC method was mainly focused on the minimization of organic solvent usage and reduction of waste generation along with occupational hazards. In addition, the proposed HPLC method utilized a simple isocratic elution with low acetonitrile percentage which is more environmentally friendly in comparison with the gradient elution methods used in the previously reported literature^16^. The analysis time of the developed RP-HPLC method was also considerably shorter (6 min) compared to the reported method (25 min)^16^, which is another important factor that contributes to the “greenness” of the method via increasing the throughput and reducing the analysis time and consequently lowering the energy consumption.

**Fig. 5:**
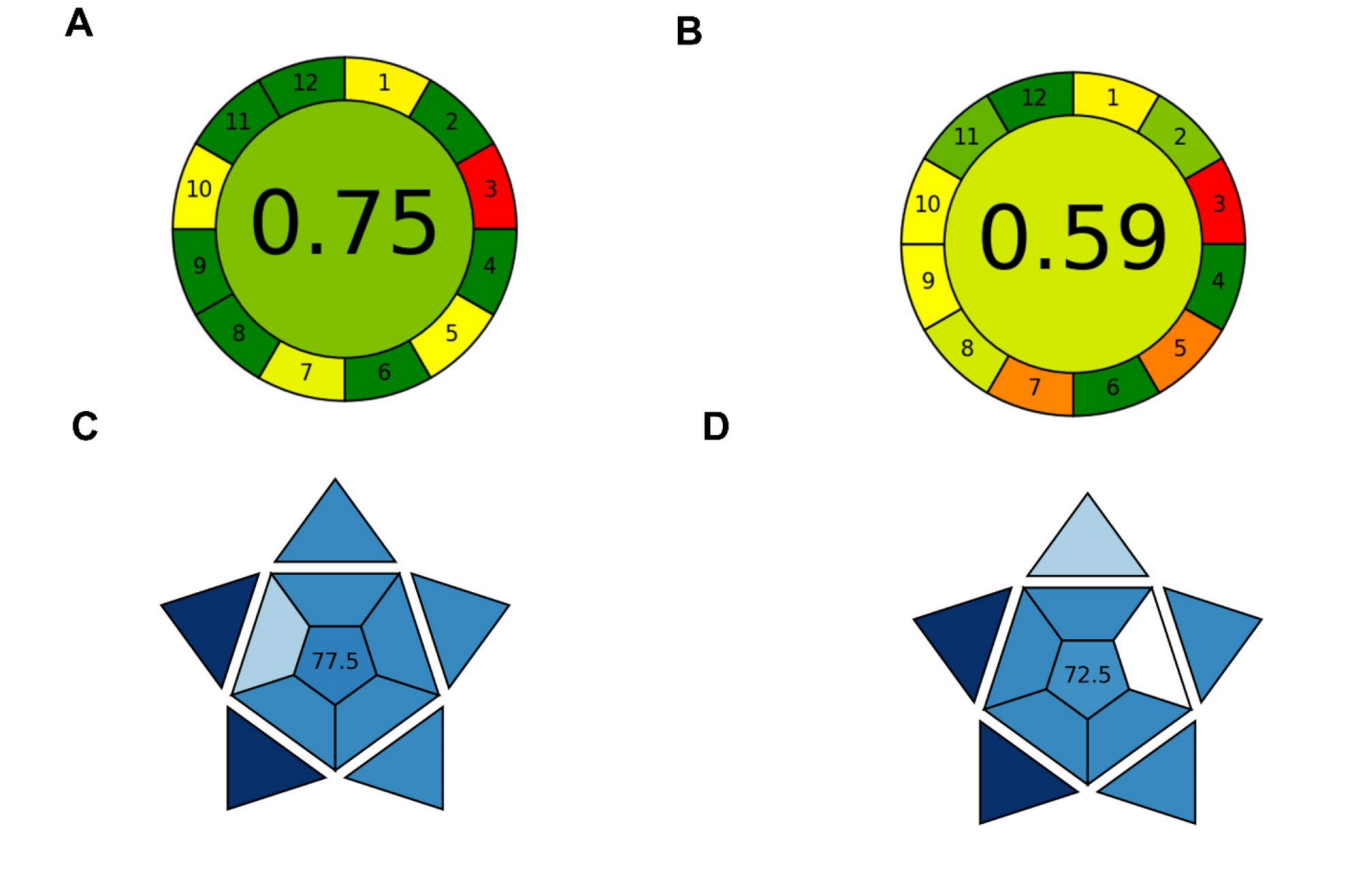
Greenness assessment of (**A**) the developed HPLC method and (**B**) the reported chromatographic method using the AGREE tool to determine their environmental impact. Blueness assessment of (**C**) the developed HPLC method and (**D**) the reported chromatographic method using the BAGI tool to determine their analytical practicability.

The environmental friendliness of the developed HPLC method was also quantitatively evaluated using a widely adopted green analytical chemistry metric: AGREE. This metric, based on the 12 principles of green chemistry, provides a score ranging from 0 to 1, where 1 represents the most environmentally friendly analytical method^33^. The developed method attained an AGREE score of 0.75 primarily due to the low percentage of acetonitrile in the mobile phase and the short run time (Fig. 5A). On the other hand, the reported HPLC method had an AGREE score of 0.59 posing a higher environmental impact and higher operational costs (Fig. 5B).

In addition to the “greenness” assessment, the “blueness” evaluation was also carried out using the BAGI tool to assess the analytical practicability of the developed HPLC method in comparison with the reported HPLC method^34^. The BAGI score considers the main factors that contribute to the analytical method’s feasibility, including cost, time efficiency, and ease of use. The BAGI score of the proposed HPLC method was 77.5 out of 100 (Fig. 5C), indicating a high level of applicability and practicability transcending the reported method with BAGI score of 72.5 (Fig. 5D) indicating its superiority in terms of analytical and operational practicability.

### Application to pharmaceutical formulations and tap water samples

To assess the applicability of the proposed HPLC method, it was evaluated for the quantitative determination of CFO, CFT, CFP and CFZ in pharmaceutical formulations. Results indicate that the developed method can accurately quantify the studied analytes in their pharmaceutical dosage forms (Table 5). Statistical comparison between the proposed, the reported^16^ and USP methods^22^ using Student’s *t*-test and *F*-test revealed no significant differences in terms of accuracy and precision, confirming the reliability of the developed method for the determination of CFO, CFT, CFP and CFZ in pharmaceutical formulations (Table 5). In addition, the applicability of the proposed method was extended to the determination of the studied cephalosporins in tap water samples. Spiked samples showed good recovery values ranging from 98 to 102% with low RSD% (below 2%), demonstrating the feasibility of the developed HPLC method for environmental monitoring of the target cephalosporins (Table 6).

**Table 5:** Quantitative analysis of CFT, CFO, CFZ and CFP in commercial pharmaceutical preparations by the proposed method and statistical comparison with the reported method and USP methods.

Drug	Method	Mean^a^	SD	RSD%	Variance	t-test^b^	F-value^b^
CFT	Developed method	99.98	1.232	1.232	1.518	–	–
	Reported method^16^	99.49	0.768	0.772	0.589	0.758	2.577
	USP method^22^	99.94	1.006	1.007	1.012	0.068	1.500
CFO	Developed method	100.02	1.068	1.068	1.140	–	–
	Reported method^16^	99.41	0.812	0.817	0.660	1.069	1.728
	USP method^22^	99.40	0.745	0.750	0.556	1.010	2.052
CFZ	Developed method	100.10	1.098	1.097	1.206	–	–
	Reported method^16^	99.73	1.501	1.505	2.252	0.441	1.868
	USP method^22^	99.36	1.186	1.194	1.407	1.020	1.167
CFP	Developed method	99.96	1.090	1.090	1.188		
	Reported method^16^	99.71	1.110	1.113	1.232	0.368	1.037
	USP method^22^	99.67	0.934	0.937	0.873	0.442	1.361

^a^Average of five determinations.

^b^Tabulated values are 2.306 and 6.338 for “t “and “F” tests, respectively.

**Table 6:** Application of the developed method to determine CFT, CFO, CFZ and CFP in tap water samples.

Amount added (µg/mL)	CFT Recovery (%) ± RSD%^a^	CFO Recovery (%) ± RSD%^a^	CFZ Recovery (%) ± RSD%^a^	CFP Recovery (%) ± RSD%^a^
10	99.15 ± 0.551	101.76 ± 1.169	100.21 ± 0.954	100.71 ± 0.585
20	101.15 ± 0.338	98.18 ± 1.011	99.83 ± 1.011	100.29 ± 0.446
30	99.64 ± 0.630	100.11 ± 0.940	101.83 ± 0.829	98.41 ± 1.283
40	101.53 ± 1.236	101.57 ± 1.274	99.81 ± 0.896	101.6 ± 1.183

^a^Average three measurements.

## Conclusion, limitations and future directions

High distribution of many infectious diseases promotes the need for using antibiotics. As cephalosporins considering being essential group and highly consumed during treatment processes, it was critical to develop a simple and fast analytical method for its determination. A Quality by Design approach was utilized to create and authenticate a cost-effective, novel, selective, fast and environmentally friendly RP-HPLC method for the analysis of four cephalosporins, (CFO, CFT, CFZ and CFP) in their pharmaceutical formulations and water samples. The Box–Behnken design was adopted to optimize chromatographic parameters with minimal experimental runs, aiming for maximum resolution and minimum total analysis time. Several green assessment tools have contended that the environmental profile of the method presented is superior compared to previously discussed approaches. The developed procedure demonstrates an outstanding environmentally friendly analytical process regarding GAC principles. Additionally, the method was validated according to guidelines of ICH Q2 (R1). The suggested approach can effectively use for regular routine analysis of the studied drugs, aiming to minimize wastage, time, and effort in the laboratory.

The major limitation of this work is that real water samples from different sources were not analyzed to evaluate the reliability of the method for environmental monitoring of the target cephalosporins. In future, the method can be further applied for the determination of the studied antibiotics in various environmental water samples. In addition, the proposed method can be combined with aptamer-based biosensors to develop fast and cost-effective screening techniques for on-site monitoring of cephalosporin residues in water bodies. Another potential future research direction could involve the assessment of the environmental impact and ecotoxicity of the target cephalosporin analytes using other analytical tools such as chemometrics coupled to advanced UV-Vis and fluorescence spectroscopic techniques, as highlighted in previous reports. Besides, evaluation of shorter HPLC columns to further enhance the greenness of the developed method by reducing solvent consumption and analysis time remains an important area of future research.

## Electronic supplementary material

Below is the link to the electronic supplementary material.

Supplementary Material 1

## Data Availability

The data presented in this study are available on request from the corresponding author.

## References

[1] Bush, K. & Bradford, P. A. β-Lactams and β-Lactamase inhibitors: an overview. *Cold Spring Harb. Perspect. Med.* **6**. 10.1101/cshperspect.a025247 (2016). 10.1101/cshperspect.a025247 PMC4968164 27329032

[2] Kim, D. et al. Structural insights for β-Lactam antibiotics. *Biomol. Ther. (Seoul)* **31**, 141–147. 10.4062/biomolther.2023.008 (2023). 36788654 10.4062/biomolther.2023.008 PMC9970833

[3] Lin, X. & Kück, U. Cephalosporins as key lead generation beta-lactam antibiotics. *Appl. Microbiol. Biotechnol.* **106**, 8007–8020. 10.1007/s00253-022-12272-8 (2022). 36401643 10.1007/s00253-022-12272-8 PMC9712332

[4] Reardon, S. Antibiotic resistance sweeping developing world. *Nature* **509**, 141–142. 10.1038/509141a (2014). 24805322 10.1038/509141a

[5] Ali Ahmed, S. M., Elbashir, A. A. & Aboul-Enein, H. Y. New spectrophotometric method for determination of cephalosporins in pharmaceutical formulations. *Arab. J. Chem.* **8**, 233–239. 10.1016/j.arabjc.2011.08.012 (2015).

[6] Roopa, K. P. & Jayanna, B. K. Spectrophotometric determination of some cephalosporins in bulk and in pharmaceutical formulations. *Anal. Chem. Lett.* **6**, 143–152. 10.1080/22297928.2016.1191970 (2016).

[7] Omar, M. A., Abdelmageed, O. H. & Attia, T. Z. Kinetic spectrophotometric determination of certain cephalosporins in pharmaceutical formulations. *Int. J. Anal. Chem.* **2009**, 596379. 10.1155/2009/596379 (2009). 10.1155/2009/596379 PMC2814136 20140078

[8] Attia, K. A. M., Nassar, M. W. I., El-Zeiny, M. B. & Serag, A. Stability indicating methods for the analysis of cefprozil in the presence of its alkaline induced degradation product. *Spectrochim. Acta Part A Mol. Biomol. Spectrosc.* **159**, 1–6. 10.1016/j.saa.2016.01.026 (2016). 10.1016/j.saa.2016.01.026 26814624

[9] Attia, K. A. M., Nassar, M. W. I., El-Zeiny, M. B. & Serag, A. Different approaches in manipulating ratio spectra applied for the analysis of cefprozil in presence of its alkaline-induced degradation product: a comparative study. *Spectrochim. Acta Part A Mol. Biomol. Spectrosc.* **145**, 289–294. 10.1016/j.saa.2015.03.038 (2015). 10.1016/j.saa.2015.03.038 25791886

[10] McWhinney, B. C. et al. Analysis of 12 beta-lactam antibiotics in human plasma by HPLC with ultraviolet detection. *J. Chromatogr. B* **878**, 2039–2043. 10.1016/j.jchromb.2010.05.027 (2010). 10.1016/j.jchromb.2010.05.027 20561826

[11] Holstege, D. M., Puschner, B., Whitehead, G. & Galey, F. D. Screening and mass spectral confirmation of beta-lactam antibiotic residues in milk using LC-MS/MS. *J. Agric. Food Chem.* **50**, 406–411. 10.1021/jf010994s (2002). 11782216 10.1021/jf010994s

[12] Chen, X. & Ye, N. Graphene oxide-reinforced hollow fiber solid-phase microextraction coupled with high-performance liquid chromatography for the determination of cephalosporins in milk samples. *Food. Anal. Methods* **9**, 2452–2462. 10.1007/s12161-016-0435-4 (2016).

[13] Rigo-Bonnin, R. et al. Development and validation of a measurement procedure based on ultra-high performance liquid chromatography-tandem mass spectrometry for simultaneous measurement of β-lactam antibiotic concentration in human plasma. *Clin. Chim. Acta* **468**, 215–224. 10.1016/j.cca.2017.03.009 (2017). 28288784 10.1016/j.cca.2017.03.009

[14] Sahebi, H., Konoz, E. & Ezabadi, A. Synthesis of DABCO-based ionic liquid functionalized magnetic nanoparticles as a novel sorbent for the determination of cephalosporins in milk samples by dispersive solid-phase extraction followed by ultra-performance liquid chromatography-tandem mass spectrometry. *New J. Chem.* **43**, 13554–13570. 10.1039/C9NJ02200G (2019).

[15] Roth, T. et al. Simultaneous determination of six antibiotics in human serum by high-performance liquid chromatography with UV detection. *Biomed. Chromatogr.* **35**, e5010. 10.1002/bmc.5010 (2021). 33119907 10.1002/bmc.5010

[16] Nemutlu, E., Kır, S., Katlan, D. & Beksaç, M. S. Simultaneous multiresponse optimization of an HPLC method to separate seven cephalosporins in plasma and amniotic fluid: application to validation and quantification of cefepime, cefixime and cefoperazone. *Talanta* **80**, 117–126. 10.1016/j.talanta.2009.06.034 (2009). 19782200 10.1016/j.talanta.2009.06.034

[17] Yu, X. et al. Distribution and persistence of cephalosporins in cephalosporin producing wastewater using SPE and UPLC–MS/MS method. *Sci. Total Environ.* **569–570**, 23–30. 10.1016/j.scitotenv.2016.06.113 (2016). 27328396 10.1016/j.scitotenv.2016.06.113

[18] Bhushan, R. & Parshad, V. Separation and identification of some cephalosporins on impregnated TLC plates. *Biomed. Chromatogr.* **10**, 258–260. https://doi.org/10.1002/(sici)1099-0801(199609)10:5<258::Aid-bmc597>3.0.Co;2-g (1996). 8879536 10.1002/(SICI)1099-0801(199609)10:5<258::AID-BMC597>3.0.CO;2-G

[19] Quesada-Molina, C., Olmo-Iruela, M. & García-Campaña, A. M. Analysis of cephalosporin residues in environmental waters by capillary zone electrophoresis with off-line and on-line preconcentration. *Anal. Methods* **4**, 2341–2347. 10.1039/C2AY25179E (2012).

[20] Wang, X., An, J., Li, J. & Ye, N. A capillary coated with a metal-organic framework for the capillary electrochromatographic determination of cephalosporins. *Microchim. Acta* **184**, 1345–1351. 10.1007/s00604-017-2131-5 (2017).

[21] Tůma, P. et al. Monitoring of amoxicilline and ceftazidime in the microdialysate of diabetic foot and serum by capillary electrophoresis with contactless conductivity detection. *Electrophoresis* **43**, 1129–1139. 10.1002/elps.202100366 (2022). 35072285 10.1002/elps.202100366

[22] The United States Pharmacopeia. 29th rev., and The National Formulary, 24th ed. (United States Pharmacopeial Convention, 2006).

[23] Vogt, F. G. & Kord, A. S. Development of quality-by-design analytical methods. *J. Pharm. Sci.* **100**, 797–812 (2011). 21280050 10.1002/jps.22325

[24] Hinkelmann, K. & Kempthorne, O. *Design and Analysis of Experiments, Volume 1: Introduction to Experimental Design* Vol. 592 (Wiley, 2007).

[25] Hammad, S. F., Habib, A. A., Kamal, A. H. & Megahed, S. M. Design of experiment-oriented development of solvent-free mixed micellar chromatographic method for concomitant determination of metronidazole and ciprofloxacin hydrochloride. *Sci. Rep.* **13**, 17352. 10.1038/s41598-023-44498-5 (2023). 37833422 10.1038/s41598-023-44498-5 PMC10575926

[26] Ganorkar, S. B. & Shirkhedkar, A. A. Design of experiments in liquid chromatography (HPLC) analysis of pharmaceuticals: Analytics, applications, implications and future prospects. *Rev. Anal. Chem.* **36**, 20160025 (2017).

[27] Kamal, A. H., Habib, A. A., Hammad, S. F. & Megahed, S. M. Quality by design paradigm for optimization of green stability indicating HPLC method for concomitant determination of fluorescein and benoxinate. *Sci. Rep.* **13**, 10471. 10.1038/s41598-023-37548-5 (2023). 37380783 10.1038/s41598-023-37548-5 PMC10307890

[28] Alanazi, Y. A. Condition optimization of eco-friendly RP-HPLC and MCR methods via Box–Behnken design and six sigma approach for detecting antibiotic residues. *Sci. Rep.* **13**, 15729. 10.1038/s41598-023-40010-1 (2023). 37735531 10.1038/s41598-023-40010-1 PMC10514345

[29] de la Guardia, M. & Garrigues, S. *Handbook of Green Analytical Chemistry* Vol. 794 (Wiley Online Library, 2012).

[30] Pena-Pereira, F., Kloskowski, A. & Namieśnik, J. Perspectives on the replacement of harmful organic solvents in analytical methodologies: a framework toward the implementation of a generation of eco-friendly alternatives. *Green Chem.* **17**, 3687–3705 (2015).

[31] Armenta, S., Garrigues, S. & de la Guardia, M. Green analytical chemistry. *TRAC Trends Anal. Chem.* **27**, 497–511 (2008).

[32] Hussein, O. G., Ahmed, D. A., Rezk, M. R., Abdelkawy, M. & Rostom, Y. Exquisite integration of quality-by-design and green analytical approaches for simultaneous determination of xylometazoline and antazoline in eye drops and rabbit aqueous humor, application to stability study. *J. Pharm. Biomed. Anal.* **235**, 115598. 10.1016/j.jpba.2023.115598 (2023). 37516064 10.1016/j.jpba.2023.115598

[33] Pena-Pereira, F., Wojnowski, W. & Tobiszewski, M. AGREE—Analytical GREEnness Metric Approach and Software. *Anal. Chem.* **92**, 10076–10082. 10.1021/acs.analchem.0c01887 (2020). 32538619 10.1021/acs.analchem.0c01887 PMC7588019

[34] Manousi, N., Wojnowski, W., Płotka-Wasylka, J. & Samanidou, V. Blue applicability grade index (BAGI) and software: a new tool for the evaluation of method practicality. *Green Chem.* **25**, 7598–7604. 10.1039/D3GC02347H (2023).

[35] Fares, M. Y., Hegazy, M. A., El-Sayed, G. M., Abdelrahman, M. M. & Abdelwahab, N. S. Quality by design approach for green HPLC method development for simultaneous analysis of two Thalassemia drugs in biological fluid with pharmacokinetic study. *RSC Adv.* **12**, 13896–13916. 10.1039/D2RA00966H (2022). 35548387 10.1039/d2ra00966h PMC9084420

[36] Patil, T. S. & Deshpande, A. S. Development of an innovative quality by design (QbD) based stability-indicating HPLC method and its validation for clofazimine from its bulk and pharmaceutical dosage forms. *Chromatographia* **82**, 579–590 (2019).

[37] ICH Guidelines.* Validation of Analytical Procedures Q2 (R1)* (ICH, 2022).

