# Supplementary material for: A quality by design HPLC method for cephalosporin analysis in pharmaceuticals and water samples with environmental impact assessment
Source: Sci Rep. 2025 Jan 2;15:33. doi: 10.1038/s41598-024-84647-y (PMC11697007; doi:10.1038/s41598-024-84647-y)
Supplement: Supplementary file 1 — Supplementary Material 1 [file 41598_2024_84647_MOESM1_ESM.docx]

**A Quality by Design HPLC Method for Cephalosporin Analysis in Pharmaceuticals and Water Samples with Environmental Impact Assessment**

**Ali Alqahtani ^a^, Taha Alqahtani ^a^, Adel Al Fatease ^b^, Enas H. Tolba ^c, *^**

^a^ Department of Pharmacology, College of Pharmacy, King Khalid University, Abha, 62529, Saudi Arabia

^b^ Department of Pharmaceutics, College of Pharmacy, King Khalid University, Abha, 62529, Saudi Arabia

^c^ Egyptian Drug Authority (EDA), Giza, 35521, Egypt

*Corresponding author email address **(Enas H. Tolba**): [nosso2@yahoo.com](mailto:nosso2@yahoo.com)

**Table S1:** Box-Behnken design data matrix and responses.

|  |  | Factor 1 | Factor 2 | Factor 3 | Response 1 | Response 2 | Response 3 | Response 4 |
| --- | --- | --- | --- | --- | --- | --- | --- | --- |
| Std | Run | A:% Acetonitrile | B:Buffer pH | C:Flow Rate | R1 | R2 | R3 | R4 |
| 1 | 12 | 5 | 4 | 1.5 | 16.47 | 5.5 | 16.51 | 7.2 |
| 2 | 15 | 15 | 4 | 1.5 | 12.33 | 2.11 | 12.01 | 5.6 |
| 3 | 11 | 5 | 6 | 1.5 | 16.1 | 5.44 | 16.33 | 7.4 |
| 4 | 13 | 15 | 6 | 1.5 | 13.21 | 2.45 | 11.55 | 5.3 |
| 5 | 4 | 5 | 5 | 1 | 16.36 | 5.13 | 16.77 | 7.3 |
| 6 | 1 | 15 | 5 | 1 | 14.33 | 4.11 | 14.88 | 6.2 |
| 7 | 3 | 5 | 5 | 2 | 11.25 | 1.55 | 10.54 | 5.4 |
| 8 | 9 | 15 | 5 | 2 | 9.01 | 1.22 | 8.34 | 4.2 |
| 9 | 10 | 10 | 4 | 1 | 15.25 | 4.44 | 15.71 | 7.1 |
| 10 | 14 | 10 | 6 | 1 | 15.31 | 4.9 | 15.22 | 7.2 |
| 11 | 2 | 10 | 4 | 2 | 10.54 | 1.37 | 9.34 | 5.3 |
| 12 | 8 | 10 | 6 | 2 | 10.85 | 1.34 | 9.25 | 5.1 |
| 13 | 7 | 10 | 5 | 1.5 | 14.03 | 3.68 | 14.6 | 6.1 |
| 14 | 5 | 10 | 5 | 1.5 | 14.31 | 3.42 | 13.99 | 6.3 |
| 15 | 6 | 10 | 5 | 1.5 | 14.15 | 3.28 | 14.3 | 6 |

R1 = resolution between ceftriaxone and cefotaxime.

R2 = resolution between cefotaxime and ceftazidime.

R3 = resolution between ceftazidime and cefoperazone.

R4= Chromatographic run time.

**Table S2**: ANOVA results for the Box-Behnken reduced quadratic model for R1 after removing non-signiﬁcant terms.

| **Source** | **Sum of Squares** | **df** | **Mean Square** | **F-value** | **p-value** |  |
| --- | --- | --- | --- | --- | --- | --- |
| **Model** | 72.48 | 3 | 24.16 | 129.08 | < 0.0001 | significant |
| A-% Acetonitrile | 15.96 | 1 | 15.96 | 85.27 | < 0.0001 |  |
| C-Flow Rate | 48.02 | 1 | 48.02 | 256.54 | < 0.0001 |  |
| C² | 8.50 | 1 | 8.50 | 45.41 | < 0.0001 |  |
| **Residual** | 2.06 | 11 | 0.1872 |  |  |  |
| Lack of Fit | 2.02 | 9 | 0.2244 | 11.37 | 0.0834 | not significant |
| Pure Error | 0.0395 | 2 | 0.0197 |  |  |  |
| **Cor Total** | 74.54 | 14 |  |  |  |  |

**Table S3**: ANOVA results for the Box-Behnken linear model for R2 after removing non-signiﬁcant terms.

| **Source** | **Sum of Squares** | **df** | **Mean Square** | **F-value** | **p-value** |  |
| --- | --- | --- | --- | --- | --- | --- |
| **Model** | 28.92 | 2 | 14.46 | 30.96 | < 0.0001 | significant |
| A-% Acetonitrile | 7.47 | 1 | 7.47 | 15.99 | 0.0018 |  |
| C-Flow Rate | 21.45 | 1 | 21.45 | 45.93 | < 0.0001 |  |
| **Residual** | 5.60 | 12 | 0.4670 |  |  |  |
| Lack of Fit | 5.52 | 10 | 0.5522 | 13.40 | 0.0714 | not significant |
| Pure Error | 0.0824 | 2 | 0.0412 |  |  |  |
| **Cor Total** | 34.52 | 14 |  |  |  |  |

**Table S4**: ANOVA results for the Box-Behnken reduced quadratic model for R3 after removing non-signiﬁcant terms.

| **Source** | **Sum of Squares** | **df** | **Mean Square** | **F-value** | **p-value** |  |
| --- | --- | --- | --- | --- | --- | --- |
| **Model** | 111.67 | 3 | 37.22 | 101.35 | < 0.0001 | significant |
| A-% Acetonitrile | 22.34 | 1 | 22.34 | 60.84 | < 0.0001 |  |
| C-Flow Rate | 78.81 | 1 | 78.81 | 214.59 | < 0.0001 |  |
| C² | 10.51 | 1 | 10.51 | 28.62 | 0.0002 |  |
| **Residual** | 4.04 | 11 | 0.3673 |  |  |  |
| Lack of Fit | 3.85 | 9 | 0.4282 | 4.60 | 0.1912 | not significant |
| Pure Error | 0.1861 | 2 | 0.0930 |  |  |  |
| **Cor Total** | 115.71 | 14 |  |  |  |  |

**Table S5**: ANOVA results for the Box-Behnken reduced linear model for R4 after removing non-signiﬁcant terms.

| **Source** | **Sum of Squares** | **df** | **Mean Square** | **F-value** | **p-value** |  |
| --- | --- | --- | --- | --- | --- | --- |
| **Model** | 12.10 | 2 | 6.05 | 64.14 | < 0.0001 | significant |
| A-% Acetonitrile | 4.50 | 1 | 4.50 | 47.69 | < 0.0001 |  |
| C-Flow Rate | 7.61 | 1 | 7.61 | 80.59 | < 0.0001 |  |
| **Residual** | 1.13 | 12 | 0.0944 |  |  |  |
| Lack of Fit | 1.09 | 10 | 0.1086 | 4.65 | 0.1898 | not significant |
| Pure Error | 0.0467 | 2 | 0.0233 |  |  |  |
| **Cor Total** | 13.24 | 14 |  |  |  |  |

**Table S6:** The fitting results of the developed models aided by BBD.

| **Model term** | **R1 response** | **R2 response** | **R3 response** | **R4 response** |
| --- | --- | --- | --- | --- |
| **R-squared** | 0.9724 | 0.8377 | 0.9651 | 0.9145 |
| **Adjusted R-squared** | 0.9648 | 0.8106 | 0.9556 | 0.9002 |
| **Predicted R-squared** | 0.9438 | 0.7154 | 0.9253 | 0.8436 |
| **Adeq Precision** | 34.58 | 17.04 | 30.74 | 25.11 |

**Table S7:** Experimental data for robustness testing of the proposed RP-HPLC method.

| Parameter | Modification | CFT R% ± RSD% ^a^ | CFO R%± RSD% ^a^ | CFZ R%± RSD% ^a^ | CFP R%± RSD% ^a^ |
| --- | --- | --- | --- | --- | --- |
| Acetonitrile % | 6 | 100.33±1.140 | 100.45±1.001 | 99.78±0.997 | 99.11±1.123 |
|  | 7 | 99.96±1.044 | 101.05±1.014 | 98.74±0.886 | 100.44±1.132 |
|  | 8 | 98.63±1.211 | 99.47±1.035 | 100.41±0.887 | 100.11±1.224 |
| Buffer pH | 5.8 | 98.69±0.997 | 99.55±0.981 | 98.46±1.005 | 99.77±0.874 |
|  | 6 | 100.25±0.945 | 101.10±0.932 | 99.98±1.117 | 100.15±0.684 |
|  | 6.2 | 100.33±0.954 | 100.24±0.884 | 99.74±1.134 | 100.02±0.687 |
| Flow rate | 1.2 | 99.25±0.974 | 100.11±1.055 | 100.78±0.992 | 99.68±0.744 |
|  | 1.3 | 100.68±0.931 | 100.85±1.044 | 100.52±0.984 | 99.14±0.765 |
|  | 1.4 | 100.36±0.777 | 99.74±1.082 | 99.65±0.777 | 100.71±0.815 |

^a^ Average three measurements


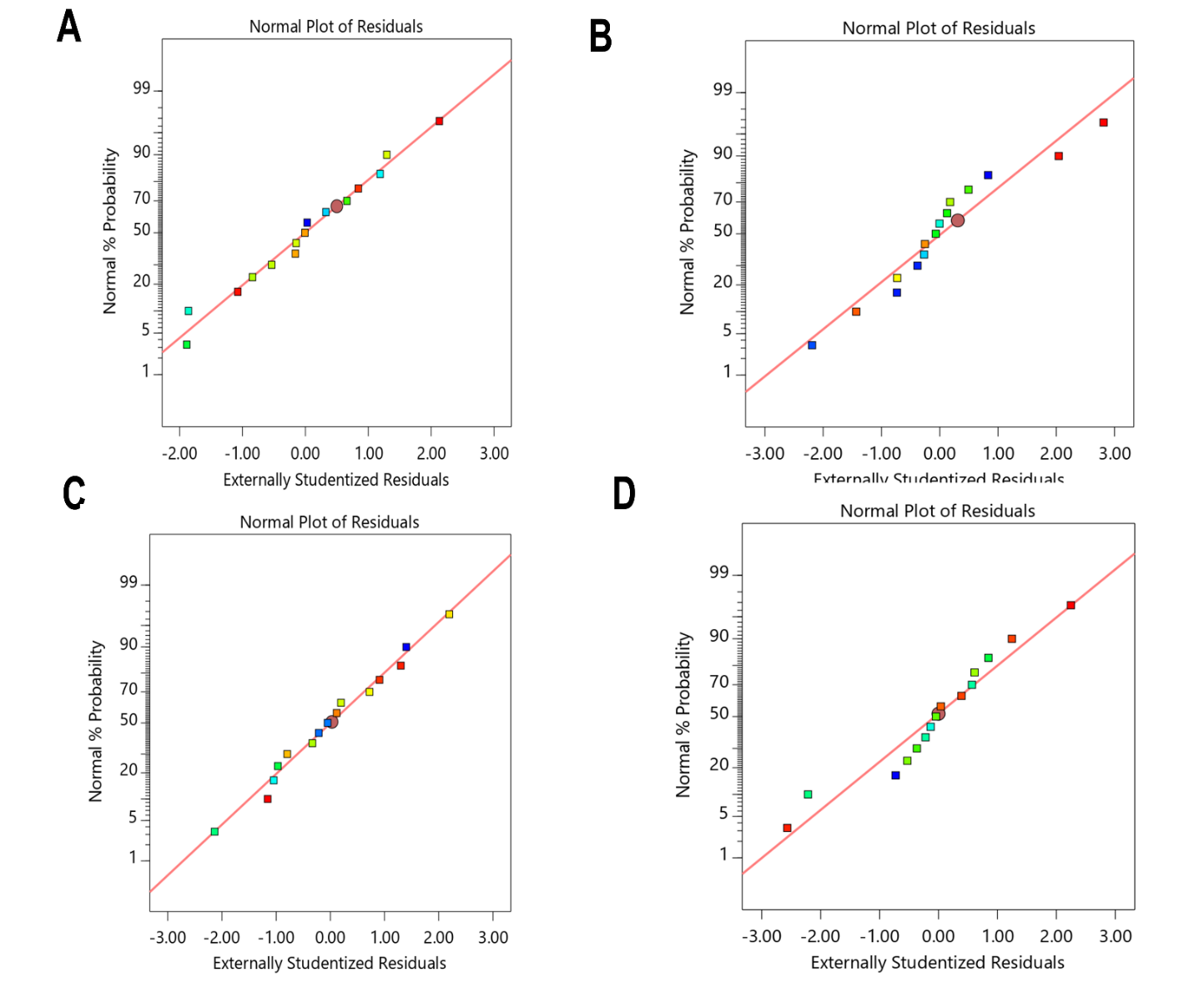


**Fig. S1:** Normal probability plot of the studentized residuals showing a straight line indicating a normal distribution of the data obtained from the developed models for (A) the resolution between ceftriaxone and cefotaxime, (B) the resolution between cefotaxime and ceftazidime, (C) resolution between ceftazidime and cefoperazone and (D) chromatographic run time.
